# Developmental Analysis of Compound Leaf Development in *Arachis hypogaea*

**DOI:** 10.3389/fpls.2022.749809

**Published:** 2022-02-10

**Authors:** Ruiqi Sun, Zhenying Peng, Shuangshuang Li, Hongyao Mei, Yiteng Xu, Wenying Yang, Zhichao Lu, Hongfeng Wang, Jing Zhang, Chuanen Zhou

**Affiliations:** ^1^The Key Laboratory of Plant Development and Environmental Adaptation Biology, Ministry of Education, School of Life Sciences, Shandong University, Qingdao, China; ^2^Institute of Crop Germplasm Resources, Shandong Academy of Agricultural Sciences, Jinan, China

**Keywords:** *Arachis hypogaea*, compound leaf pattern, pentafoliate, tetrafoliate, cytokinin, *KNOXI*

## Abstract

Leaves are the primary photosynthetic structures, while photosynthesis is the direct motivation of crop yield formation. As a legume plant, peanut (*Arachis hypogaea*) is one of the most economically essential crops as well as an important source of edible oil and protein. The leaves of *A. hypogaea* are in the tetrafoliate form, which is different from the trifoliate leaf pattern of *Medicago truncatula*, a model legume species. In *A*. *hypogaea*, an even-pinnate leaf with a pair of proximal and distal leaflets was developed; however, only a single terminal leaflet and a pair of lateral leaflets were formed in the odd-pinnate leaf in *M. truncatula*. In this study, the development of compound leaf in *A. hypogaea* was investigated. Transcriptomic profiles revealed that the common and unique differentially expressed genes were identified in a proximal leaflet and a distal leaflet, which provided a research route to understand the leaf development in *A. hypogaea*. Then, a naturally occurring mutant line with leaf developmental defects in *A. hypogaea* was obtained, which displayed a pentafoliate form with an extra terminal leaflet. The characterization of the mutant indicated that cytokinin and class I *KNOTTED-LIKE HOMEOBOX* were involved in the control of compound leaf pattern in *A. hypogaea*. These results expand our knowledge and provide insights into the molecular mechanism underlying the formation of different compound leaf patterns among species.

## Introduction

Leaves are the major organs of plants for photosynthesis and serve as their prime mediator with the environment above the soil surface. There are many forms of leaves, but they can be classified as simple leaves or compound leaves according to the number of blades. Simple leaves supported by a petiole have a single blade unit, whereas compound leaves attached to a rachis by leaflets have multiple blade units. Genetic evidence shows that the occurrence of leaflet primordia during compound leaf development is similar to that of simple leaf primordia (Hasson et al., 2010).

Compared to simple leaves, compound leaves suffered much less pressure or resistance to wind and rain, which significantly improves the ability of the plant to adapt to harsh conditions (Vogel, 2009). Moreover, the development of compound leaves requires the establishment of leaf properties and polarity according to different development patterns, rather than the simple addition of single leaves. Compound leaves were initiated from the periphery zone (PZ) of the pluripotent shoot apical meristem (SAM). Leaf morphogenesis occurs in three successive stages: (1) initiation of the leaf primordium which is recruited from the PZ of the SAM, (2) primary morphogenesis, i.e., the primordium of each principal component of the compound leaf was divided, while the adaxial-abaxial, mediolateral, and proximal-distal axes were established, and (3) expansion and secondary morphogenesis, i.e., intercalation growth occurred throughout the entire leaf blade, resulting in an overall expansion of leaf area in multiple directions (Dengler and Tsukaya, 2001; Du et al., 2018).

Auxin plays a crucial role in leaves. In shoot, auxin efflux carrier PINFORMED1 (PIN1) actively directs the transportation and distribution of auxin (Reinhardt et al., 2003). The inhibition of auxin activity or transportation resulted in simplified leaves in *Cardamine*, tomato, and pea (Bar and Ori, 2015). Moreover, the downregulation of *IAA9*, a distinct subfamily of *Aux/IAA* genes, results in simple leaves in tomato (Wang et al., 2005; Zhang et al., 2007). In *Medicago truncatula*, *MtPIN10/SLM1* loss-of-function mutant shows the impaired auxin distribution, resulting in increased terminal leaflets and reduced lateral leaflets (Zhou et al., 2011). In addition, shoot apices treated with the auxin transport inhibitor *N*-1-naphthylphthalamic acid (NPA) lead to a pin-like structure without leaves (Reinhardt et al., 2000). The external application of indole-3-acetic acid (IAA) to the apices in NPA-treated and *pin1* mutant restores leaf formation (Reinhardt et al., 2000). These findings demonstrate that auxin is tightly correlated with leaf development.

During compound leaf development, class I KNOTTED-LIKE HOMEOBOX (KNOXI) family is required for leaflet formation. For example, the KNOXI homeobox transcription factor TKN2 antagonizes *CLAUSA* (*CLAU*) in regulating the morphogenesis-differentiation balance of the compound leaf development in tomato (Israeli et al., 2021). *CLAU* is a negative regulator of *KNOXI* genes, and *clau* mutant showed excessively divided leaves (Bar et al., 2016). In *M. truncatula*, class M KNOX protein FCL1 encodes a truncated KNOX that lacks the homeodomain. FCL1 plays a key role in boundary separation, and *fcl1* mutants show fused leaflets (Peng et al., 2011). The plant hormone cytokinin (CK) acts downstream of KNOXI proteins to maintain the prolonged morphogenetic activity of the leaf margin (Shani et al., 2010). In addition, the shoot and leaf development is retarded in CK-deficient mutants in *Arabidopsis*, such as reduced shoot growth rates, reduced size of SAM, and reduced cell production in the leaves, indicating that CK is a positive regulator in cell division (Werner et al., 2003).

Leguminosae is the third largest family of angiosperms and contains about 19,000 species in about 750 genera. Legumes are a good source of protein that contains high iron, folate, potassium, and magnesium. They also contain beneficial fats and soluble and insoluble fiber. Most plants of legumes have typically compound leaves, while a few subfamilies have simple leaves. The compound leaf development in legumes has been studied in several species, such as *M. truncatula*, *Lotus japonicus*, and *Vigna radiata* (Wang et al., 2008, 2013; Jiao et al., 2019). Peanut (*Arachis hypogaea*) is one of the most economically essential legume crops (Xu et al., 2021). The leaf in *A. hypogaea* is tetrafoliate with a pair of proximal and distal leaflets, which is different from that in *M. truncatula* and *L. japonicus*. However, the regulation mechanism of compound leaf pattern in *A. hypogaea* is largely unknown. In this study, we focused on the ontogeny of leaf development and characterized a mutant with pentafoliate leaf form in *A. hypogaea*. The possible developmental mechanism of tetrafoliate leaf form in *A. hypogaea* was proposed.

## Materials and Methods

### Plant Materials and Growth Condition

A cultivated peanut variety Fenghua-1 was used as a wild type. The plants with pentafoliate leaf form were a naturally occurring mutant line in the genetic background of the variety Fenghua-1. The peanut plants were grown in soil with a photoperiod of 16-h day/8-h night, a temperature of 22°C, and a relative humidity of 70% in a growth chamber.

### Scanning Electron Microscopy

Shoot apices were collected from 4 weeks post-germination of wild-type and mutant plant. Plant tissues were fixed in a fixative solution (3.0% glutaraldehyde in 25 mM phosphate buffer, pH 7.0, and 0.1% Trixon-100) by vacuum infiltration for 10 min and then incubated in 4°C overnight. On the following day, the tissues were dehydrated in a series of graded ethanol (30, 50, 60, 70, 85, 95, and 100% three times), each lasting for a minimum of 20 min. A critical-point drier was used to dry the ethanol in liquid CO_2_ to remove the alcohol. The tissues were mounted on aluminum stubs, dissected under a stereoscopic microscope, and sputtered with gold. Tissue samples were then examined using Tecnai G2 F20 Scanning Electron Microscope (SEM) at an accelerating voltage of 5 kV (FEI).

### RNA Extraction and Real-Time PCR Analysis

Total RNA from different frozen tissues was extracted using EASYspin Plus Complex Plant RNA Kit (Aidlab). The quality of RNA was measured by NanoDrop 2000 spectrophotometer (Thermo Fisher Scientific) to detect the concentration and integrity using RNA Reverse Transcription Kit (Roche). Real-time PCR was performed using SYBR Green (Roche), while data acquisition and analysis were performed using Bio-Rad CFX Connect TM sequence detection system. Three biological replicates were applied in the assay, and each biological replicate was technically replicated three times. Gene expression levels were calculated and normalized by the arithmetic mean with *AhADH3* used as housekeeping genes (Brand and Hovav, 2010). The single-factor ANOVA method was used to estimate if the difference in gene expression level is significant.

### Transcriptomic Analysis

For the transcriptomic analysis of proximal and distal leaflets, the newly emerged folded leaflets and petioles at the early developmental stage were harvested from 40-day-old wild-type plants. For the transcriptomic analysis of leaf development in the mutant, the shoot buds were harvested from 40-day-old wild-type and pentafoliate mutant plants. Three biological replicates of each sample were prepared. Total RNA of each sample was extracted, and all samples were sequenced on a BGISEQ-500 platform at the BGI Genomics Institute (BGI-Shenzhen). Differentially expressed genes (DEGs) were determined using an upregulated/downregulated more than twofold and a false discovery rate (FDR) < 0.01. The FDR method was used to adjust the hypergeometric test of the *p*-value to evaluate the enrichment degree of the Gene Ontology (GO) items and the KEGG pathway. The heatmap was created by Helm software (Heatmap Illustrator, version 1.0).

### Phenotypic Analysis

For leaf morphology analysis, wild-type and mutant plants were photographed by the Nikon D300 camera. Nikon SMZ 1500 stereomicroscope (Nikon) was used to image the close-up view of the leaf. At least 10 samples were observed from each experiment.

### Phylogenetic Analyses

The candidate KNOXI proteins were identified in *Arabidopsis thaliana*, *M. truncatula*, and *A. hypogaea*. Amino acid sequences were downloaded from the websites of Phytozome and NCBI. All confirmed amino acids sequences were aligned using the ClustalW program. The neighbor-joining phylogenetic trees were constructed using the MEGA7 software, with 1,000 bootstrap iterations.

### Cytokinin Treatment

Of note, 4-week-old wild-type plants growing in soil were sprayed with a solution containing 0.1, 0.25, and 0.5 mM 6-benzyladenine (6-BA) with 0.01% Tween 20, respectively. The pentafoliate mutants were sprayed with lovastatin (Lov) at a concentration of 0.01 and 0.05 μM with 0.01% Tween 20. The same concentration of Tween 20 was applied in the mock treatments. Twelve plants were used in each group for analysis. 6-BA and Lov were sprayed every 2 days for five times, and the leaf phenotypes were analyzed with three biological replicates.

## Results

### Ontogeny of Compound Leaf Development in *Arachis hypogaea*

**FIGURE 1:**
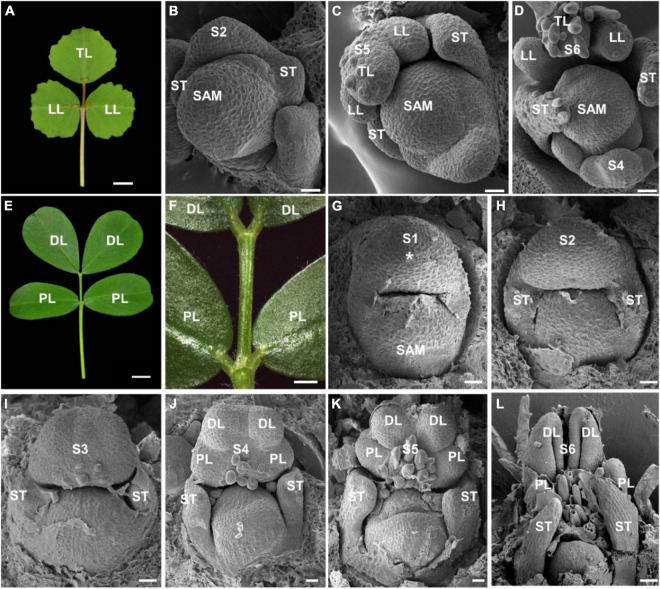
The ontogeny of compound leaf development in *Medicago truncatula* and *Arachis hypogaea*. **(A)** The mature leaf morphology of *M. truncatula*. **(B–D)** Different stages of compound leaf development in *M. truncatula*. **(B)** Stage 2. A pair of stipule primordia (ST) were initiated from leaf primordium. **(C)** Stage 5. The common leaf primordium differentiated into a pair of lateral leaflet primordia (LL) and a terminal leaflet primordium (TL). **(D)** Stage 6. Trichomes were developed on terminal leaflet primordium. **(E,F)** Fully expanded compound leaf of *A. hypogaea*. A close-up view is shown in panel **(F)**. **(G–L)** The Scanning Electron Microscopy (SEM) analysis of the ontogeny of leaf development in *A. hypogaea*. **(G)** Stage 1. Leaf primordium (asterisk) initiates and grows to enwrap the SAM. **(H)** Stage 2. A pair of stipule primordia (ST) were initiated from the proximal end of the leaf primordium. **(I)** Stage 3. The boundaries formed between the common leaf primordium and stipule leaflet primordia. **(J)** Stage 4. A pair of proximal leaflet primordia (PL) and a pair of distal leaflet primordia (DL) were formed. **(K)** Stage 5. Trichomes were developed. **(L)** Stage 6. The leaflet primordia became folded. Bars = 0.5 cm in panels **(A,E)**, 20 μm in panels **(B–D)**, 0.2 cm in panel **(F)**, and 50 μm in panel **(G–L)**.

To investigate the compound leaf development in peanut, we first compared the leaf patterns between *M. truncatula* and *A. hypogaea*. As a model legume species, *M. truncatula* has a typical trifoliate leaf pattern (Figure 1A). The degree of compoundness in *M. truncatula* is much simpler than other compound leaf species, which develops a pair of lateral leaflets and a terminal leaflet (Wang et al., 2008). The SEM analysis showed that a common leaf primordium and a pair of stipule primordia were initiated at stage 2 (Figure 1B). At stage 5, a pair of lateral leaflet primordia and one terminal leaflet primordium were observed (Figure 1C). Subsequently, the leaflet primordia and trichomes were developed at stage 6 (Figure 1D).

The leaves of *A. hypogaea* plant are in tetrafoliate form, displaying the pinnately compound leaf with two pairs of leaflets (Figure 1E). It is noted that unique even-pinnate leaflets were formed in *A. hypogaea*, which are significantly different from the odd-pinnate leaflets in *M. truncatula* (Figure 1F). SEM observation showed that a common leaf primordium was formed from the flank of SAM at stage 1 (Figure 1G). At stage 2, a pair of stipule primordia have emerged at the proximal end of the common leaf primordium (Figure 1H). Then, the boundaries between the two stipules and the common leaf primordium were established at stage 3 (Figure 1I). At stage 4, the common leaf primordium was differentiated into a pair of proximal leaflet and distal leaflet primordia (Figure 1J). Subsequently, at stage 5, the proximal and distal leaflet primordia separated away from each other so that the boundaries were established, and spherical trichomes were initiated (Figure 1K). At stage 6, the proximal leaflet and distal leaflet primordia became folded. Trichomes were differentiated further as tubular trichomes. Finally, a pair of proximal leaflets and distal leaflets were formed (Figure 1L).

### Transcriptomic Analysis Between the Developing Proximal Leaflet and Distal Leaflet

**FIGURE 2:**
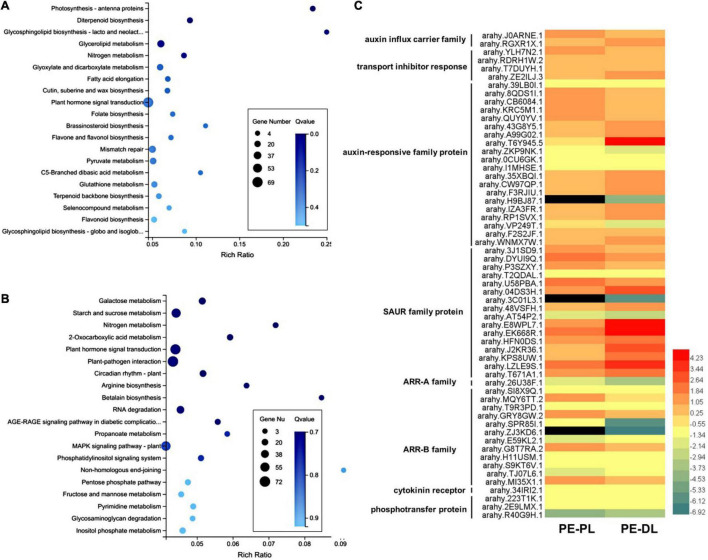
Analysis of differentially expressed genes (DEGs) in proximal and distal leaflets in *A. hypogaea*. **(A)** KEGG enrichment analysis of DEGs in distal leaflets. **(B)** KEGG enrichment analysis of DEGs in proximal leaflets. **(C)** A heatmap of DEGs enriched in auxin and cytokinin (CK) signaling transduction pathway between proximal and distal leaflets. PL, proximal leaflet, DL, distal leaflet, PE, petiole.

To compare the proximal leaflet and distal leaflet of *A. hypogaea*, the RNA-seq transcriptomic analysis was performed using newly developed folded proximal leaflet, distal leaflet, and petiole at the vegetative stage as materials. Genes with more than twofold expression changes and *p*-values < 0.05 were identified as DEGs. Compared with the petiole, 4,617 upregulated DEGs and 8,885 downregulated DEGs were identified in the distal leaflet, while 6,504 upregulated DEGs and 8,959 downregulated DEGs were identified in the proximal leaflet (Supplementary Tables 1, 2). Then, we compared the DEGs in a proximal leaflet and a distal leaflet using petiole as a control. The results showed that 2,354 DEGs were exclusively detected in developing distal leaflets. The KEGG analysis showed that plant hormone signal transduction, glycerolipid metabolism, and mismatch repair were significantly enriched, in which 69 DEGs were involved in plant hormone signal transduction (Figure 2A). Among 69 DEGs, 33 DEGs were involved in auxin signaling pathways, while 4 DEGs were involved in the CK pathway. Moreover, the exclusive DEGs in the proximal leaflet were enriched in plant hormone signal transduction, starch and sucrose metabolism, plant-pathogen interaction, and MAPK signaling pathway (Figure 2B). Among them, 67 DEGs were involved in plant hormone signal transduction, including 18 DEGs in auxin signaling pathways and 13 DEGs in the CK pathway. Furthermore, in auxin signal transduction pathways, there were 143 DEGs in common in both proximal and distal leaflets, while 18 and 33 DEGs existed exclusively in the proximal and distal leaflet, respectively. In CK signal transduction pathways, there were 46 DEGs in common in both proximal and distal leaflets, while 13 and 4 DEGs existed exclusively in the proximal and distal leaflet, respectively. In addition, the DEGs enriched in auxin and CK pathway were compared between proximal leaflet and distal leaflet, and part of them displayed the different expression levels (Figure 2C and Supplementary Table 3). These results imply that auxin and CK signal transduction may play different roles in the developing proximal and distal leaflets. These data indicate that most genes related to auxin and CK signal transduction pathways play similar roles during the development of both proximal leaflet and distal leaflet.

### Identification of the Mutant Line With Pentafoliate Leaf Form in *Arachis hypogaea*

**FIGURE 3:**
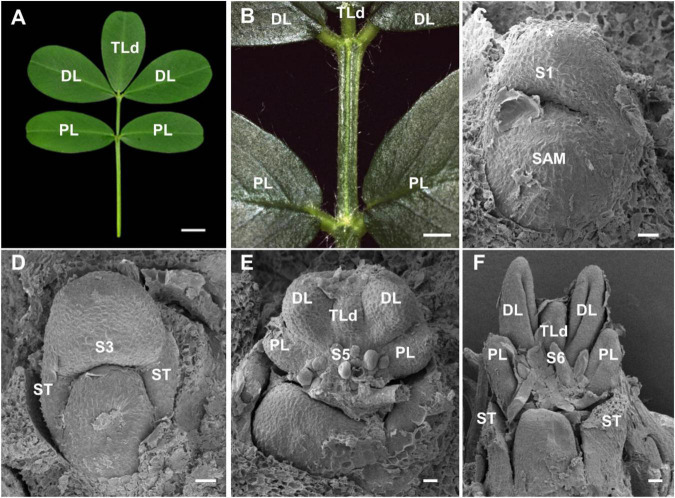
Characterization of pentafoliate mutant in *A. hypogaea*. **(A)** About 46.1% of leaves showed pentafoliate leaf form in the mutant. **(B)** The close-up view of the leaf in panel **(A)**. **(C,D)** The leaf primordia were emerged at stage 1 **(C)**, and the stipule primordia were formed at stage 3 **(D)**. **(E,F)** Distally oriented terminal leaflet (TLd) primordium was emerged and developed between the distal leaflet primordia (DL) at stage 5 **(E)** and stage 6 **(F)**. Bars = 0.5 cm **(A)**, 0.2 cm in panel **(B)**, and 50 μm in panels **(C–F)**.

During the cultivation of cultivated peanut variety Fenghua-1, a naturally occurring mutant with leaf defects was isolated in the same genetic background. About 46.1% of leaves in mutant showed pentafoliate leaf form, and this phenotype could be stably inherited. Compared with wild type, a distally oriented terminal leaflet (TLd) was developed between two distal leaflets in the mutant (Figures 3A,B). SEM analysis showed that leaf primordium was initiated at stage 1, and a pair of stipule primordia had emerged at stage 3, which is similar to those in wild type (Figures 3C,D). At stage 5, the TLd primordium has emerged between a pair of distal leaflet primordia in the mutant (Figure 3E). At stage 6, the boundaries were established between the TLd and distal leaflet primordia, but the development of TLd was slower than that of the distal leaflet (Figure 3F).

### Transcriptomic Analysis Between Wild Type and Mutant

**FIGURE 4:**
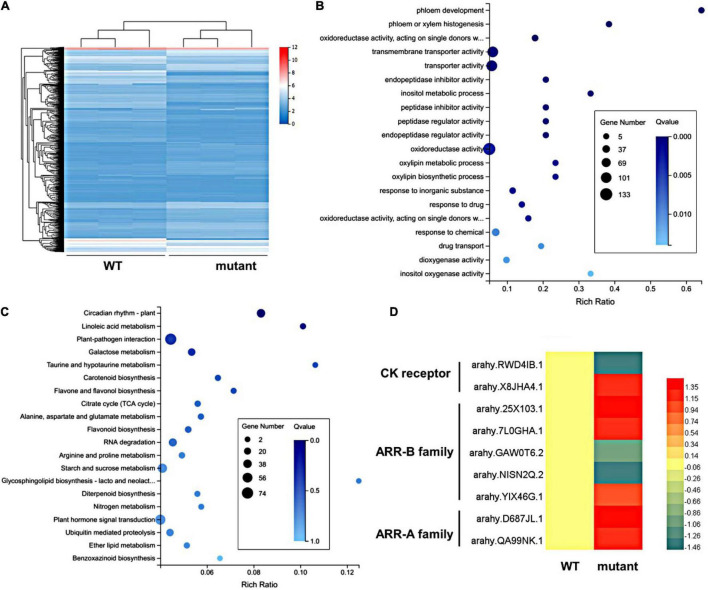
The DEG analysis in wild type and mutant. **(A)** The heatmap of Fragments Per Kilobase of transcript per Million mapped reads (FPKM) values of 2,252 DEGs between the wild type and the mutant shoot buds in three biological replicates. **(B)** Gene Ontology (GO) enrichment analysis of DEGs. **(C)** KEGG enrichment analysis of DEGs. **(D)** The heatmap of expression levels of CK-related DEGs.

To gain insight into the developmental mechanism of the pentafoliate mutant, the transcriptomic analysis was performed using RNA-seq. The transcriptomes of shoot apices were acquired from wild type and mutant, and a total of 2,252 DEGs were identified (Supplementary Table 4). Among them, 1,150 DEGs were upregulated, and 1,102 DEGs were downregulated (Figure 4A). The GO term enrichment analysis showed that the most enriched GO terms were transmembrane transporter activity, transporter activity, and oxidoreductase activity (Figure 4B). The KEGG analysis showed that plant-pathogen interaction, starch and sucrose metabolism, and plant hormone signal transduction were significantly enriched (Figure 4C). There are 61 DEGs involved in plant hormone signal transduction, such as auxin, CK, and gibberellin (GA) signaling and other hormone pathways (Supplementary Table 5). CK can alter leaf differentiation by changing its concentration (Bar et al., 2016). In the transcriptomes of the CK signaling pathway, 9 DEGs were enriched, including 2 DEGs in the CK receptor, 5 DEGs in the B-ARR family, and 2 DEGs in the A-ARR family (Figure 4D and Supplementary Table 6). B-ARRs act as key players and positive regulators in CK signal transduction, while A-ARRs are the targets of B-ARRs (To et al., 2004; Mason et al., 2005). Most of them were upregulated in the mutant, implying that increased CK activity may be related to the additional TLd in the mutant.

### Cytokinin Plays a Key Role in the Compound Leaf Development in *Arachis hypogaea*

**FIGURE 5:**
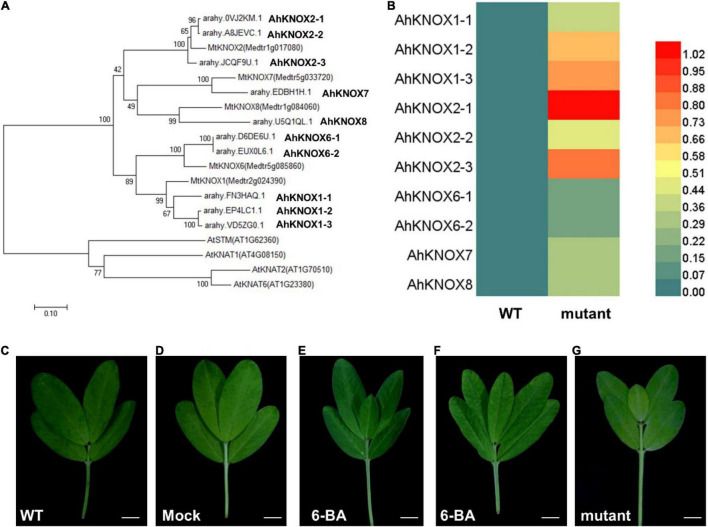
*AhKNOXI* and CK are involved in the compound leaf pattern of *A. hypogaea.* **(A)** Phylogenetic analysis of *KNOXI* genes in *A. thaliana*, *M. truncatula*, and *A. hypogaea*. **(B)** A heatmap analysis of *AhKNOXI* genes expression in wild type and mutant. **(C)** Adult leaf of wild type. **(D)** Adult leaf of wild type treated with the same concentration of Tween 20 as control. **(E,F)** Adult leaves of wild type treated with 6-benzyladenine (6-BA). **(G)** Adult leaf of the pentafoliate mutant. Bar = 1 cm **(C–G)**.

The KNOXI family has been reported to be involved in promoting the extended morphogenesis in leaves in many plant species (Barth et al., 2009; Rast-Somssich et al., 2015). In previous studies, the gene regulatory network in leaf development was identified in tomato and two related wild species, indicating that KNOX homeobox genes are located in the bottleneck position (Ichihashi et al., 2014). In addition, Class I *KNOX* genes in *M. truncatula* were isolated (Di Giacomo et al., 2008; Zhou et al., 2014) and increased the expression levels of *STM/BP-like KNOXI* genes in plants exhibited higher-order leaflets, suggesting their conserved roles in regulating leaf complexity in *M. truncatula* (Zhou et al., 2014). The phylogenetic analysis of KNOXI protein from *A. thaliana*, *M. truncatula*, and *A. hypogaea* indicated that 10 AhKNOXI proteins existed in *A. hypogaea* (Figure 5A). According to the relationship of the KNOXI proteins in *M. truncatula*, we named AhKNOXI in *A. hypogaea*. To investigate whether *AhKNOXI* genes are involved in the leaf development of mutant, their expression levels derived from the data of transcriptomic profiles were analyzed. The results showed that all of them were upregulated in pentafoliate mutant plants, while *AhKNOX1-3*, *AhKNOX2-1*, and *AhKNOX2-3* were significantly upregulated than other *AhKNOXI* (Figure 5B). Previous reports showed that KNOXI proteins are able to activate CK biosynthesis in SAM, and CK can partially rescue the loss-of-KNOXI function in *A. thaliana* (Yanai et al., 2005; Sakamoto et al., 2006). Therefore, these data imply that the increased expression levels of *AhKNOXI* genes probably result in the CK-related DEGs in the mutant.

To further investigate the relationship between CK and leaf pattern in *A. hypogaea*, wild-type plants were treated with 6-BA at a concentration of 0.1, 0.25, and 0.5 mM for five times at 2-day intervals, and the same concentration of Tween 20 was applied as a control. The observations showed that 0.5 mM 6-BA treatment could induce the additional terminal leaflet between two distal leaflets. About 10.8% of wild-type leaves were changed from tetrafoliate form to pentafoliate form, which mimicked the mutant phenotype (Figures 5C–G). These data indicate that exogenous CK treatment is sufficient for increasing leaf complexity in *A. hypogaea*. Lov is an effective inhibitor of the mevalonate pathway and has been used to eliminate the biosynthesis of CK (Hartig and Beck, 2005). However, the mutants treated with Lov at a concentration of 0.01 and 0.05 μM for five times at 2-day intervals did not recover the defects (Supplementary Figures 1A–D). These observations imply that the CK signal transduction pathway, instead of the CK biosynthetic pathway, was probably defective in the mutant.

## Discussion

Leaves are vital to plants for their ability to process photosynthesis. The diversity of leaf shapes has evolved to adapt to the environment, by maximizing the ability to absorb sunlight (Liu et al., 2021). Most legume species have compound leaf structures. *M. truncatula* is composed of a pair of lateral leaflets and a single terminal leaflet, which is similar to *Glycine max* and *L. japonicus* (Wang et al., 2008, 2013; Jiao et al., 2019). However, the compound leaf pattern in *A. hypogaea* is different from those species, which is the tetrafoliate form with a pair of proximal and distal leaflets. It is surprising that 2,354 DEGs were exclusively involved in the distal leaflet, and 2,315 DEGs were only shown in the proximal leaflet, implying that the development between proximal leaflet and distal leaflet is different. The KEGG pathway analysis showed that DEGs were significantly enriched in plant hormone signal transduction, which is coincidental with their important role in leaf development. These data imply that different hormone-related genes are involved in the formation of both proximal leaflet and distal leaflet.

The investigation of mutants helps us better understand the developmental mechanism of leaves. In this study, we discovered a naturally occurring mutant plant with an extra terminal leaflet in *A. hypogaea*, leading to the transformation of the compound leaf pattern from tetrafoliate to pentafoliate form. Transcriptomic and KEGG pathway analyses suggest that plant hormone signal transduction plays a crucial role in regulating leaf development in the mutant, in which CK signal transduction-related genes are changed. CK is essential for multiple developmental processes in plants (Kieber and Schaller, 2018), such as organ initiation, SAM size, and phyllotaxis (Giulini et al., 2004; Leibfried et al., 2005; Besnard et al., 2014). *Arabidopsis* CK biosynthesis gene *ISOPENTENYL TRANSFERASE7* (*IPT7*) and CK degradation gene *CYTOKININ OXIDASE3* (*CKX3*) under the control of *FILpro* lead to super-compound leaves and simplified leaves in tomato, indicating that CK regulates the morphogenesis of compound leaves, and different CK levels result in the alterations in leaf complexity (Shani et al., 2010).

In previous studies, CK biosynthesis is positively regulated by KNOXI proteins (Sakamoto et al., 2006), and CK acts downstream of KNOXI proteins to maintain the prolonged morphogenetic activity (Shani et al., 2010). KNOXI proteins are required in the leaf primordia to produce a dissected leaf form in compound leaf development (Hay and Tsiantis, 2006). In tomato, the overexpression of *KNOXI* leads to increase leaf complexity (Hareven et al., 1996). It has been reported that CK and *KNOXI* have a positive correlation (Hay et al., 2004). In this study, the expression levels of multiple *AhKNOXI* genes were increased in mutant plants, implying that CK level may be correlated with the extended morphogenesis in leaves of mutants. According to this, the increasing CK level in wild type is able to induce the extra terminal leaflet, which is similar to that in the mutant. Therefore, CK is probably a key regulator in the control of compound leaf patterns in *A. hypogaea*. However, mutant treated with Lov did not recover its defects, implying that CK signal transduction instead of the biosynthetic pathway is probably defective in the mutant. Moreover, the transcriptomic data showed the DEGs enriched in several plant hormone pathways such as auxin and GA signaling pathways, suggesting that these hormones also probably contribute to the mutant phenotype.

The relationship between lateral leaflet and terminal leaflet development has been reported in several studies. In *M. truncatula*, *sgl1* mutant exhibits simple leaves due to the failure of the initiation of lateral leaflet primordium, indicating that *SGL1* plays important roles in the lateral leaflet development (Wang et al., 2008). Moreover, *slm1* mutant shows multiple terminal leaflets and reduced lateral leaflet number associated with lower *SGL1* expression in *M. truncatula* (Zhou et al., 2011). Previous reports also show that the morphogenetic activity of the terminal leaflet is suppressed by the BLH protein PINNA1 in *M. truncatula* (He et al., 2020). Additionally, *M. truncatula* Cys(2) His(2) zinc finger transcription factor PALM1 binds to and downregulates the expression of *SGL1*, and the loss-of-function mutant shows five leaflets clustered at the leaf tip (Chen et al., 2010). The characteristics of these mutants indicate that the distinct developmental domains between the terminal and lateral leaflet formation existed. In *A. hypogaea*, exogenous CK treatment in wild type results in the additional terminal leaflet between a pair of distal leaflets, instead of the proximal leaflet, indicating that the developmental response to CK is different between proximal leaflet and distal leaflet.

## Conclusion

In this study, we analyzed the ontogeny of compound leaf development in *A. hypogaea*. The transcriptomic profiles between different leaflets are clarified, providing the potential gene networks for regulating leaf development in legumes. The characterization of a pentafoliate mutant suggested that CK plays a critical role in compound leaf patterns in *A. hypogaea*. Leaf area affects the efficiency of photosynthesis and thus influences yield. Therefore, the investigation of the developmental mechanism of compound leaves in peanut may contribute to improve its production by the molecular design of leaf patterning.

## Data Availability Statement

The original contributions presented in the study are publicly available. This data can be found here: National Center for Biotechnology Information (NCBI) BioProject database under accession number GSE180915.

## Author Contributions

RS, SL, and CZ designed the research. RS, ZP, SL, and JZ performed the experiments and analyzed the data. HM, YX, and WY assisted in analysis experiments and revised the manuscript. ZL and HW contributed to the new analytical tools. RS and CZ wrote the manuscript. All authors contributed to the article and approved the submitted version.

## Conflict of Interest

The authors declare that the research was conducted in the absence of any commercial or financial relationships that could be construed as a potential conflict of interest.

## Publisher’s Note

All claims expressed in this article are solely those of the authors and do not necessarily represent those of their affiliated organizations, or those of the publisher, the editors and the reviewers. Any product that may be evaluated in this article, or claim that may be made by its manufacturer, is not guaranteed or endorsed by the publisher.
